# Supplementary material for: The fecal microbiota of Thai school-aged children associated with demographic factors and diet
Source: PeerJ. 2022 Apr 20;10:e13325. doi: 10.7717/peerj.13325 (PMC9034706; doi:10.7717/peerj.13325)
Supplement: File S5 [file peerj-10-13325-s004.docx]

**Supplementary File 3:** A detailed explanation of multivariate statistical approaches used in the study of the gut microbiota of 127 Thai school-aged children.

1. **Multiple Factor Analysis (MFA)**

In this study, the MFA was constructed by integration of host variables (dietary behaviors or anthropological factors) and abundance of gut microbiota to determine the influence of multiple factors on variations of gut bacterial profiles of children The MFA was performed using FactorMine R version 2.3 (Lê et al., 2008). The importance of variables was described by the contributions (loading) to the component.

- 1. **Multiple sets of variables used in the MFA analysis**

The variables are organized in groups as follows:

- **Diets** – a categorical variable specifying dietary behaviors. Dietary behaviors were labeled as follows: HEB (healthy eating behavior), FV (fruits and vegetables), HSFB (high sugar foods and beverages), HSF (high salt foods), and HFF (high fat foods).
  - Note: For the MFA concerning dietary behaviors, two subjects were excluded from the analysis (ID: BH300 and BH240) as they were only a single sample in the categories. BH300 had HSF and FV in a high-risk level and BH240 also had HSF in a high-risk level. Therefore, the total number of subjects included in the MFA of diets was 125.
- **Anthropological factors** – the total number of subjects included in the MFA of host factors was 126. One subject was excluded from the analysis (ID: BH202) as BMI-for-age was severe thinness and was only a single sample in this category.
  - **Gender** – a categorical variable specifying gender was labeled as ‘Male’ and ‘Female’
  - **Age tertile** – a categorical variable specifying age tertile was assigned as age_A (≤ 8.05 years; n = 32), age_B (8.05 < years < 11.06; n = 61), and age_C (≥ 11.06 years; n = 34). Age groups were defined according to interquartile range (IQR: 25%, 50%, and 75%).
  - **BMI-for-age** – a categorical variable specifying BMI-for-age was assigned as severe thinness, thinness, normal weight, overweight, and obese.
  - **Ethnicity** – a categorical variable specifying five ethnic groups: Akha, Chinese, Lahu, Thai, and Thai Yai.
  - **Birth mode** – a categorical variable specifying birth delivery mode: vaginal delivery and cesarean section.
  - **Feeding type** – a categorical variable specifying feeding types: breastfeeding, formula feeding, and mixed feeding.
- **Total bacteria** – a quantitative variable specifying the abundance of total bacteria in feces.
- **Phylum** – a group of quantitative variables specifying the abundance of gut bacteria at phylum level: Firmicutes and Bacteroidetes.
- **Class** – a group of quantitative variables specifying the abundance of gut bacteria at class level: *Gamma*-*Proteobacteria*.
- **Genus** – a group of quantitative variables specifying the abundance of gut bacteria at genus level: *Prevotella*, *Roseburia*, *Ruminococcus*, *Faecalibacterium*, *Bacteroides*, *Akkermansia*, *Bifidobacteria*, and *Lactobacillus*.
  1. **Data normalization (the MFA weighting)**

The variances in the variables of the data set were computed based on PCA (for quantitative variables) and MCA (for categorical variables). To balance the influence of each group of variables in the MFA, each group of variables was normalized by dividing them by the first eigenvalue obtained from its PCA. So, the same weighting value was applied to variables in the same groups.

- 1. **Graphic outputs**

Factoextra version 1.0.7 (Kassambara & Mundt, 2020) was used to extract and visualize the MFA results. Three outputs included ‘Variable Factor Map’ (the correlation between the groups of variables and the dimensions), ‘Correlation Circle plot for quantitative variables’ (the contributions of bacterial taxa to the dimensions), and ‘Individual plot’ (variations of individual profiles colored according to categorical variables and the cluster indicated by the 95% confidence ellipses).

1. **Partial Least Squares Discriminant Analysis (PLS-DA)**

We performed PLS-DA for variable classification using the mixOmics package version 6.12.2 (Rohart et al., 2017). The goal was to determine the most relevant feature (bacterial taxa) for the discrimination among classes (dietary habits, gender, age, BMI-for-age, ethnicity, birth mode, and feeding type).

- 1. **Factors for multiclass classification**
     - - **Gender –** a categorical variable specifying gender was labeled as ‘Male’ and ‘Female’
       - **Age tertile** – a categorical variable specifying age tertile was assigned as age_A (≤ 8.05 years; n = 32), age_B (8.05 < years < 11.06; n = 61), and age_C (≥ 11.06 years; n = 34). Age groups were defined according to interquartile range (IQR: 25%, 50%, and 75%).
       - **BMI-for-age** – a categorical variable specifying BMI-for-age was assigned as severe thinness, thinness, normal weight, overweight, and obese.
       - **Ethnicity** – a categorical variable specifying five ethnic groups: Akha, Chinese, Lahu, Thai, and Thai Yai.
       - **Birth mode** – a categorical variable specifying birth delivery mode: vaginal delivery and cesarean section.
       - **Feeding type** – a categorical variable specifying feeding types: breastfeeding, formula feeding, and mixed feeding.
       - **Note:** the total number of subjects included in PLS-DA of dietary habits and BMI-for-age was 125 and 126, respectively (as mentioned in the MFA section). For PLS-DA models of other host factors, no subject was excluded (n = 127).
  2. **Features for a supervised analysis with PLS-DA**
- The abundance of gut bacteria including total bacteria, Firmicutes, Bacteroidetes, *Gamma*-*Proteobacteria*, *Prevotella*, *Roseburia*, *Ruminococcus*, *Faecalibacterium*, *Bacteroides*, *Akkermansia*, *Bifidobacteria*, and *Lactobacillus*.
- PLS-DA conditions:
  - All bacterial taxa were kept in X-loadings (n = 12).
  - Canonical mode was applied for evaluating the associations between bacterial taxa and classes with 100 iterations of the algorithms.
  - The abundance of gut bacteria was standardized to zero means and unit variances.
  1. **Graphic outputs for PLS-DA**
     - - **Sample plots (plotIndiv):**

Individual representation was based on the X-data set (the abundance of gut bacteria) and the Y-data set (categorical variables). The plot included the 95% confidence ellipses. The explained variance was calculated by each component related to the X variate.

- - - - **Loading plots (plotLoadings):**

The loading plot represents the loading weights of the gut bacteria on each component of the sample plot. The importance of the bacteria contributing to the dimension runs from bottom to top. Colors represented the class that had the highest (contrib = ‘max’) or lowest (contrib = ‘min’) median abundance of gut bacteria.

**References**

Kassambara, A., & Mundt, F. (2020). factoextra: Extract and Visualize the Results of Multivariate Data Analyses. *R Package Version 1.0.7*. https://cran.r-project.org/package=factoextra

Lê, S., Josse, J., & Husson, F. (2008). FactoMineR: An R package for multivariate analysis. *Journal of Statistical Software*, *25*, 1–18.

Rohart, F., Gautier, B., Singh, A., & Lê Cao, K.-A. (2017). mixOmics: An R package for ‘omics feature selection and multiple data integration. *PLOS Computational Biology*, *13*(11), e1005752-. https://doi.org/10.1371/journal.pcbi.1005752
