# Supplementary material for: The fecal microbiota of Thai school-aged children associated with demographic factors and diet
Source: PeerJ. 2022 Apr 20;10:e13325. doi: 10.7717/peerj.13325 (PMC9034706; doi:10.7717/peerj.13325)
Supplement: Table S1 [file peerj-10-13325-s008.docx]

**Supplementary table** Primer pairs targeting bacterial 16S rRNA genes

| **Target microorganism^a^** | **Primer sequence (5′—>3′)^b,c^** | **Product size (bp)** | **Ta** | **Efficiency (%)** | **Reference** |
| --- | --- | --- | --- | --- | --- |
| Total bacteria  (Universal 180) | F: AAACTCAAAKGAATTGACGG  R: CTCACRRCACGAGCTGAC | 180 | 60 | 91 | (Bacchetti De Gregoris et al. 2011) |
| Firmicutes (P) | F: TGAAACTYAAAGGAATTGACG  R: ACCATGCACCACCTGTC | 200 | 64 | 87 | (Bacchetti De Gregoris et al. 2011) |
| Bacteroidetes (P) | F: CRAACAGGATTAGATACCCT  R: GGTAAGGTTCCTCGCGTAT | 240 | 64 | 74 | (Bacchetti De Gregoris et al. 2011) |
| ɣ-*Proteobacteria* (C) | F: TCGTCAGCTCGTGTYGTGA  R: CGTAAGGGCCATGATG | 170 | 53 | 74 | (Bacchetti De Gregoris et al. 2011) |
| *Prevotella* spp. (G) | F: GGTTCTGAGAGGAAGGTCCCC  R: TCCTGCACGCTACTTGGCTG | 267 | 56 | 81 | (Stevenson and Weimer 2007) |
| *Roseburia* (G) | F: GCGGTRCGGCAAGTCTGA  R: CCTCCGACACTCTAGTMCGAC | 80 | 55 | 86 | (Walker et al. 2005; Ramirez-Farias et al. 2008) |
| *Ruminococcus* (G) | F: GGCGGCYTRCTGGGCTTT  R: CCAGGTGGATWACTTATTGTGTTAA | 156 | 63 | 68 | (Ramirez-Farias et al. 2008) |
| *Faecalibacterium* (G) | F: GGAGGAAGAAGGTCTTCGG  R: AATTCCGCCTACCTCTGCACT | 247 | 63 | 79 | (Wang et al. 1996; Ramirez-Farias et al. 2008) |
| *Bacteroides* (G) | F: GAAGGTCCCCCACATTG  R: CGCKACTTGGCTGGTTCAG | 103 | 64 | 89 | (Bartosch et al. 2004; Ramirez-Farias et al. 2008) |
| *Akkermansia* (G) | F: CAGCACGTGAAGGTGGGGAC  R: CCTTGCGGTTGGCTTCAGAT | 349 | 60 | 91 | (Collado et al. 2007) |
| *Bifidobacteria* (G) | F: TCGCGTCYGGTGTGAAAG  R: GGTGTTCTTCCCGATATCTACA | 601 | 64 | 79 | (Matsuki et al. 2002; Rinttila et al. 2004) |
| *Lactobacillus* spp. (G) | F: AGCAGTAGGGAATCTTCCA  R: CACCGCTACACATGGAG | 341 | 56 | 84 | (Walter et al. 2001; Heilig et al. 2002) |

Note: ^a^P=phylum, C=class and G=genus; ^b^F and R represent forward and reverse primers, respectively.

^c^Degenerate base covering: R = A/G; Y = T/C; M = A/C; W = A/T; K = T/G

**References:**

Bacchetti De Gregoris T, Aldred N, Clare AS, et al. (2011) Improvement of phylum- and class-specific primers for real-time PCR quantification of bacterial taxa. J Microbiol Methods 86:351–356. https://doi.org/https://doi.org/10.1016/j.mimet.2011.06.010

Bartosch S, Fite A, Macfarlane GT, et al. (2004) Characterization of Bacterial Communities in Feces from Healthy Elderly Volunteers and Hospitalized Elderly Patients by Using Real-Time PCR and Effects of Antibiotic Treatment on the Fecal Microbiota. Appl Environ Microbiol 70:3575–3581. https://doi.org/10.1128/AEM.70.6.3575-3581.2004

Collado MC, Derrien M, Isolauri E, et al. (2007) Intestinal Integrity and *Akkermansia muciniphila* , a Mucin-Degrading Member of the Intestinal Microbiota Present in Infants, Adults, and the Elderly. Appl Environ Microbiol 73. https://doi.org/10.1128/AEM.01477-07

Heilig HGHJ, Zoetendal EG, Vaughan EE, et al. (2002) Molecular Diversity of Lactobacillus spp. and Other Lactic Acid Bacteria in the Human Intestine as Determined by Specific Amplification of 16S Ribosomal DNA. Appl Environ Microbiol 68:114–123. https://doi.org/10.1128/AEM.68.1.114-123.2002

Matsuki T, Watanabe K, Fujimoto J, et al. (2002) Development of 16S rRNA-Gene-Targeted Group-Specific Primers for the Detection and Identification of Predominant Bacteria in Human Feces. Appl Environ Microbiol 68:5445–5451. https://doi.org/10.1128/AEM.68.11.5445-5451.2002

Ramirez-Farias C, Slezak K, Fuller Z, et al. (2008) Effect of inulin on the human gut microbiota: stimulation of *Bifidobacterium adolescentis* and *Faecalibacterium prausnitzii*. Br J Nutr 101:541–550. https://doi.org/10.1017/S0007114508019880

Rinttila T, Kassinen A, Malinen E, et al. (2004) Development of an extensive set of 16S rDNA-targeted primers for quantification of pathogenic and indigenous bacteria in faecal samples by real-time PCR. J Appl Microbiol 97:1166–1177. https://doi.org/10.1111/j.1365-2672.2004.02409.x

Stevenson DM, Weimer PJ (2007) Dominance of Prevotella and low abundance of classical ruminal bacterial species in the bovine rumen revealed by relative quantification real-time PCR. Appl Microbiol Biotechnol 75:165–174. https://doi.org/10.1007/s00253-006-0802-y

Walker AW, Duncan SH, McWilliam Leitch EC, et al. (2005) pH and Peptide Supply Can Radically Alter Bacterial Populations and Short-Chain Fatty Acid Ratios within Microbial Communities from the Human Colon. Appl Environ Microbiol 71. https://doi.org/10.1128/AEM.71.7.3692-3700.2005

Walter J, Hertel C, Tannock GW, et al. (2001) Detection of Lactobacillus, Pediococcus, Leuconostoc, and Weissella Species in Human Feces by Using Group-Specific PCR Primers and Denaturing Gradient Gel Electrophoresis. Appl Environ Microbiol 67:2578–2585. https://doi.org/10.1128/AEM.67.6.2578-2585.2001

Wang RF, Cao WW, Cerniglia CE (1996) PCR detection and quantitation of predominant anaerobic bacteria in human and animal fecal samples. Appl Environ Microbiol 62. https://doi.org/10.1128/aem.62.4.1242-1247.1996
