# Supplementary figures and images for: The fecal microbiota of Thai school-aged children associated with demographic factors and diet
Source: PeerJ. 2022 Apr 20;10:e13325. doi: 10.7717/peerj.13325 (PMC9034706; doi:10.7717/peerj.13325)

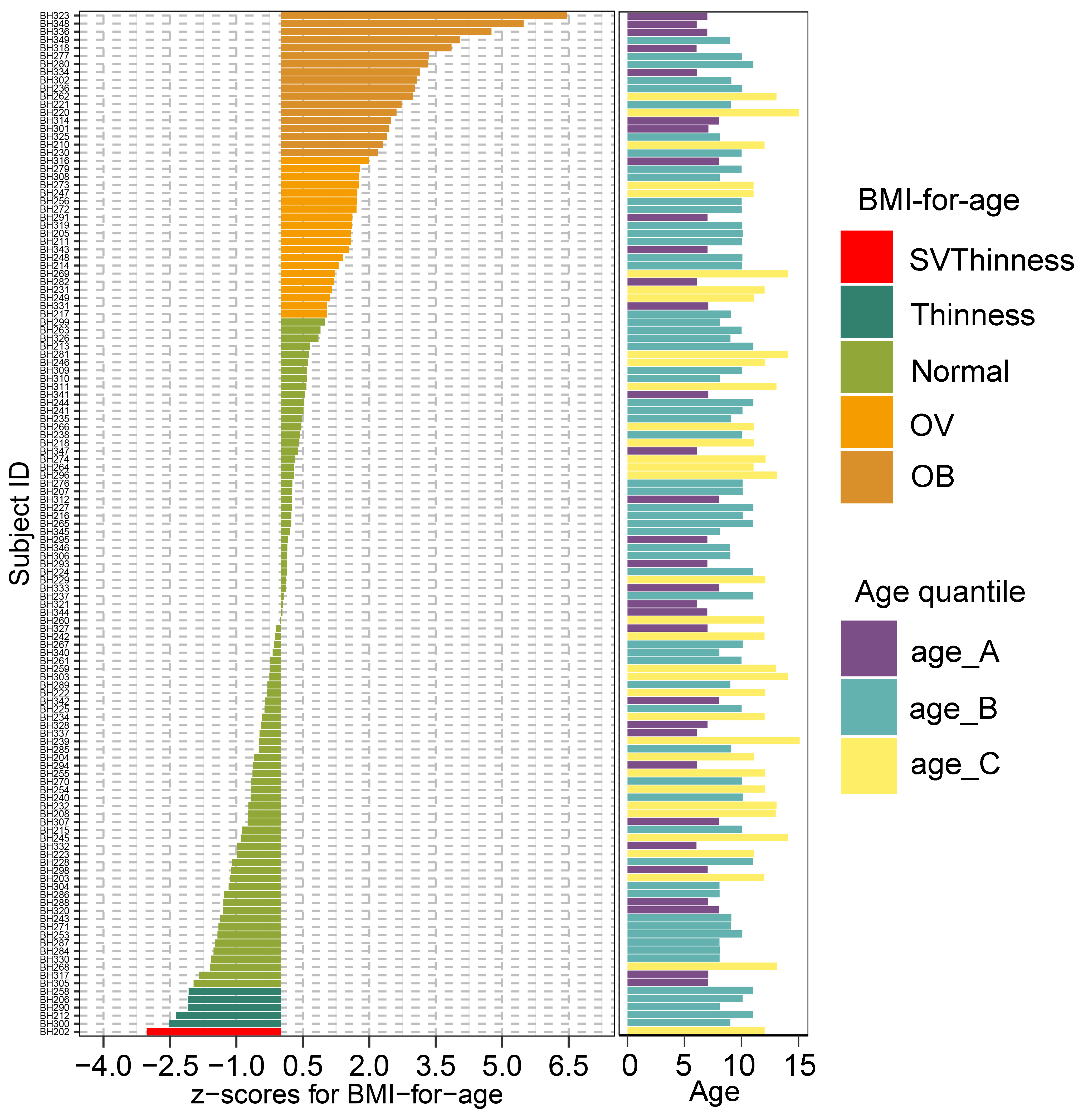

Supplement: Figure S1 — An age variable was grouped by age tertile. BMI z-score cut-off points were based on WHO Multicentre Growth Reference Study Group (2006); SVThinness (severe thinness) < −3SD, Thinness ≥−3SD to < −2SD, Normal ≥−2SD to ≤+1SD, OV (overweight) > +1SD to ≤+2SD, OB (obese) > +2SD. Age tertile(corresponding to 25 %, 50 %, and 75 %); age_A ≤ 8.05 years, age_B 8.05 < age < 11.06 years, age_C ≥ 11.06 years. [file peerj-10-13325-s009.png]

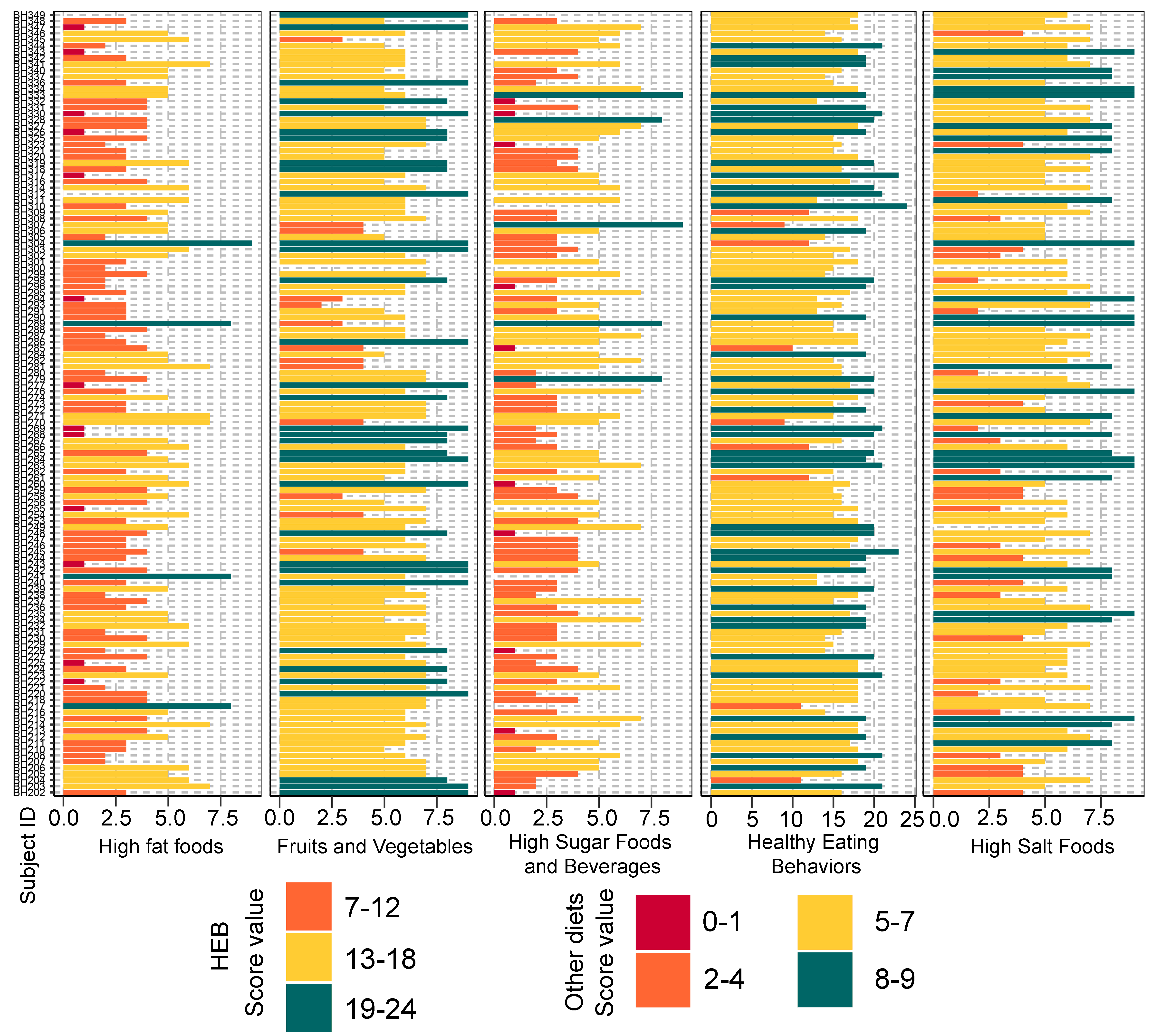

Supplement: Figure S2 — Dietary behaviors (HFF, HSFB, HSF, FV) were categorized as follows: (8–9 = low risk, 5–7 = low to moderate risk, 2–4 = moderate to high risk, 0–1 = high risk). Dietary behavior scores were then summed into a composite score for HEB (19–24 = low risk, 13–18 = low to moderate risk, 7–12 = moderate to high risk, 0–6 = high risk). The levels of risk (low, low to moderate, moderate to high, high) refer to increased frequency of consumption (e.g., of sugar, fat, salt). The highest risk refers to an individual eating the most of these three categories. The scores are in reverse direction for healthy eating behaviors as well as fruits and vegetables, in which the highest frequency of consumption is associated with the lowest risk. HEB—healthy eating behaviors. [file peerj-10-13325-s010.png]

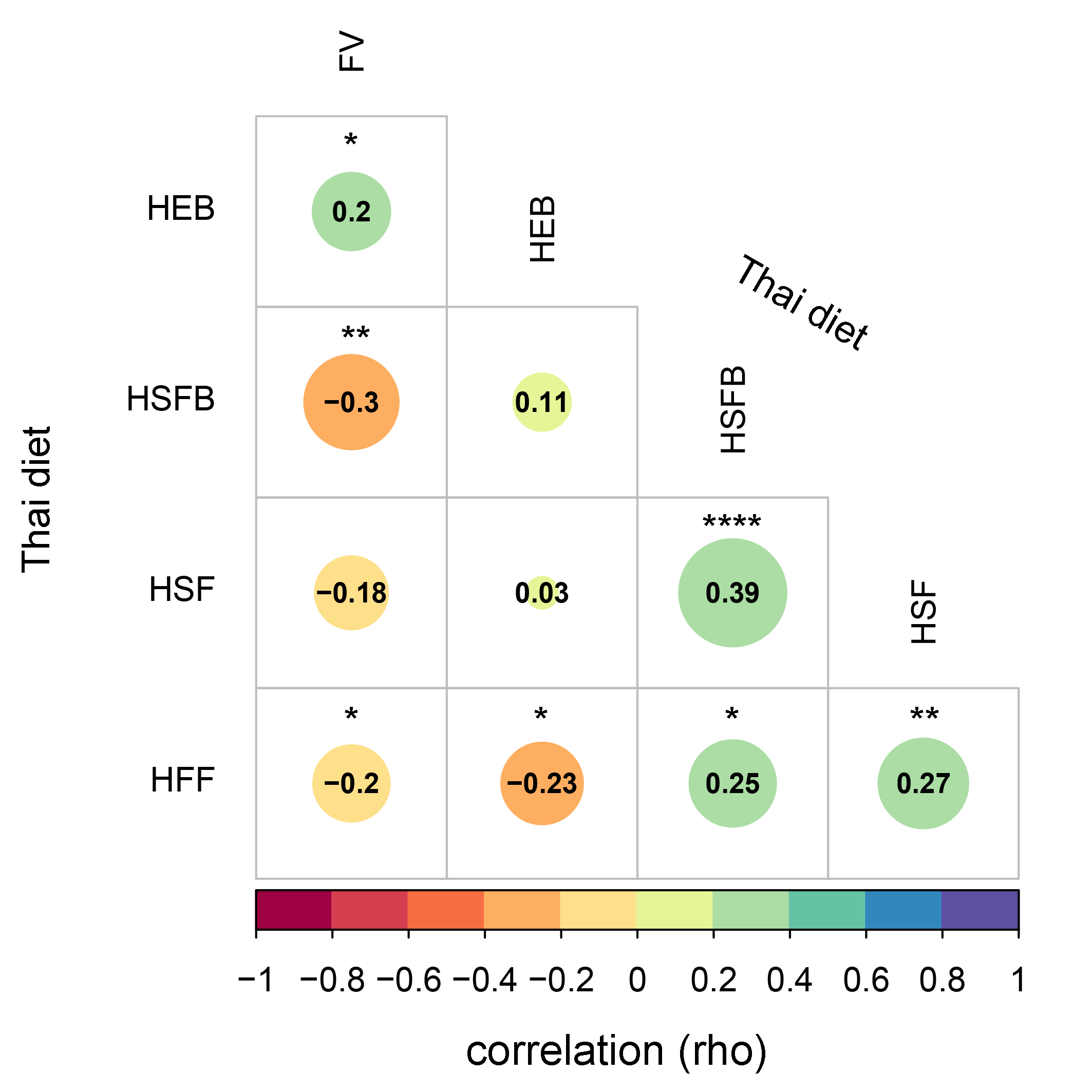

Supplement: Figure S3 — The relationship, indicated by the color bar, was assessed by using Spearman correlation coefficient strength (Rho) with size of circle denoting the strength of the correlation between variables. Asterisk indicate a significant difference correlation coefficient between variables after multiple testing correction (****q < 0.0001, ***q < 0.001, **q < 0.01, *q < 0.05; Benjamini-Hochberg p-value correction method). HEB—Healthy eating behavior; HSFB—high sugar foods and beverages; HSF—high salt foods; HFF—high fat foods; FV—fruits and vegetables. [file peerj-10-13325-s011.png]

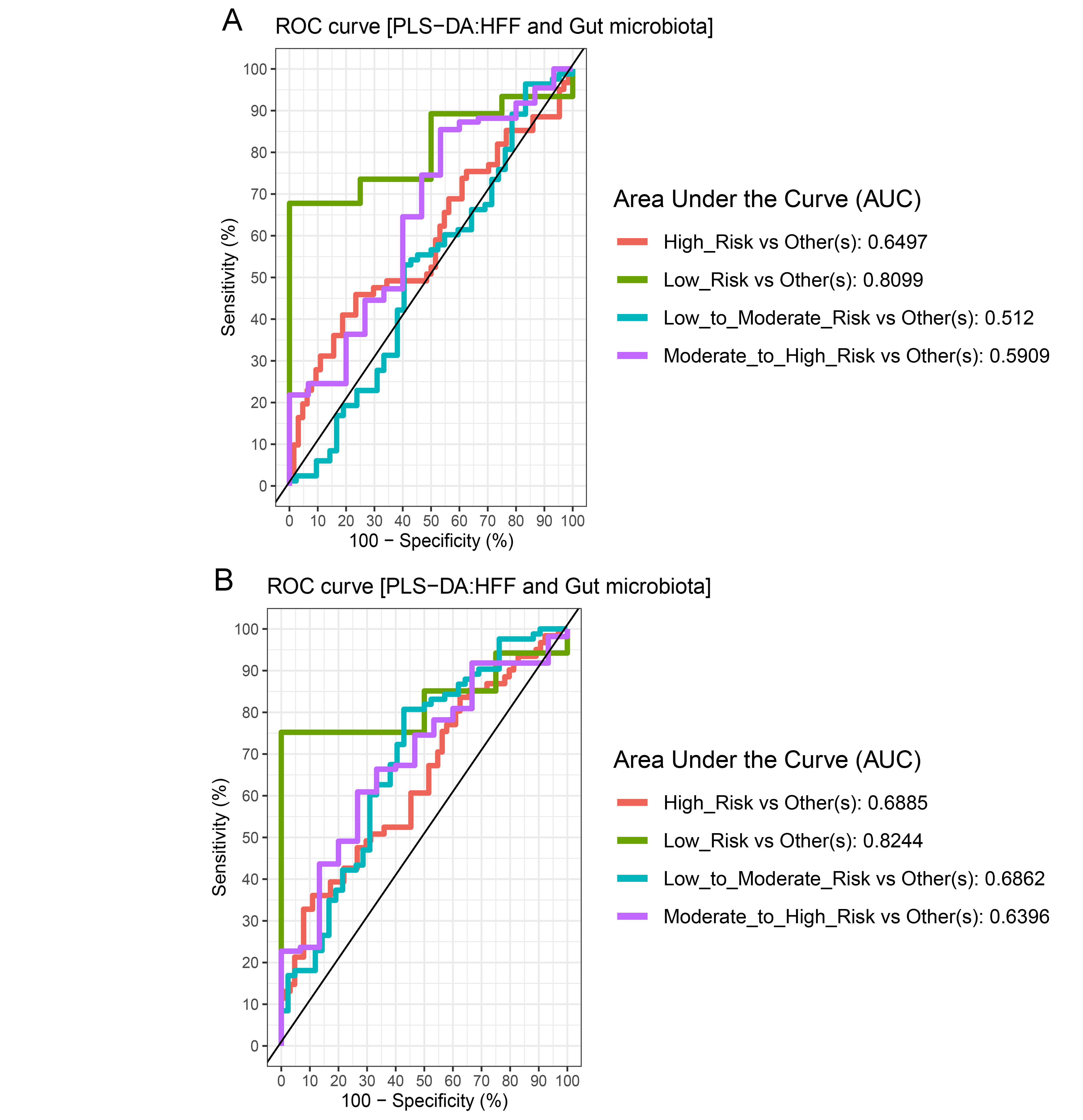

Supplement: Figure S4 — (A: component 1, B: component 2). AUC was calculated and compared between one class versus the others using Wilcoxon test. [file peerj-10-13325-s012.png]

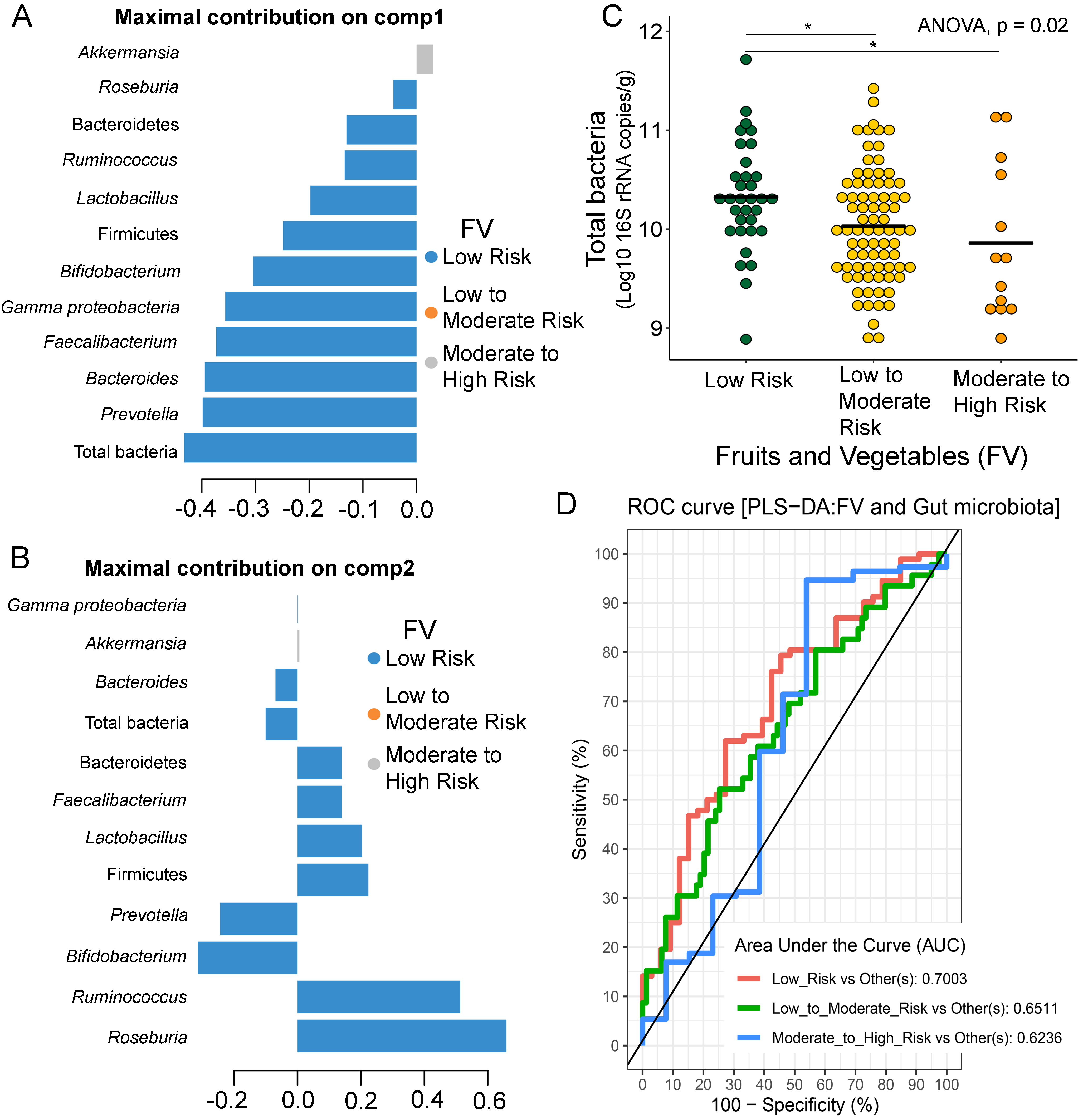

Supplement: Figure S5 — Discriminant analysis demonstrating variable selection (microbiota taxa) for which the median (method = ’median’) is maximum in component 1 (A) and component 2 (B). Horizontal bars indicate each bacterial taxon assigned to FV behaviors and their length corresponds to the loading weight. The importance of the bacteria contributing to the dimension runs from the bottom to the top of the figure. (C) Boxplots showing normalized microbiota abundances based on log10 qPCR 16S rRNA copy number per gram of feces. Asterisks indicates a significant difference in microbiota abundance among FV consumption (*q < 0.05, Tukey’s HSD test). (D) The plots of ROC curves of PLS-DA classification for gut microbiota in children with FV behaviors in component 2. AUC was calculated and compared between one class versus the others using Wilcoxon test. [file peerj-10-13325-s013.png]

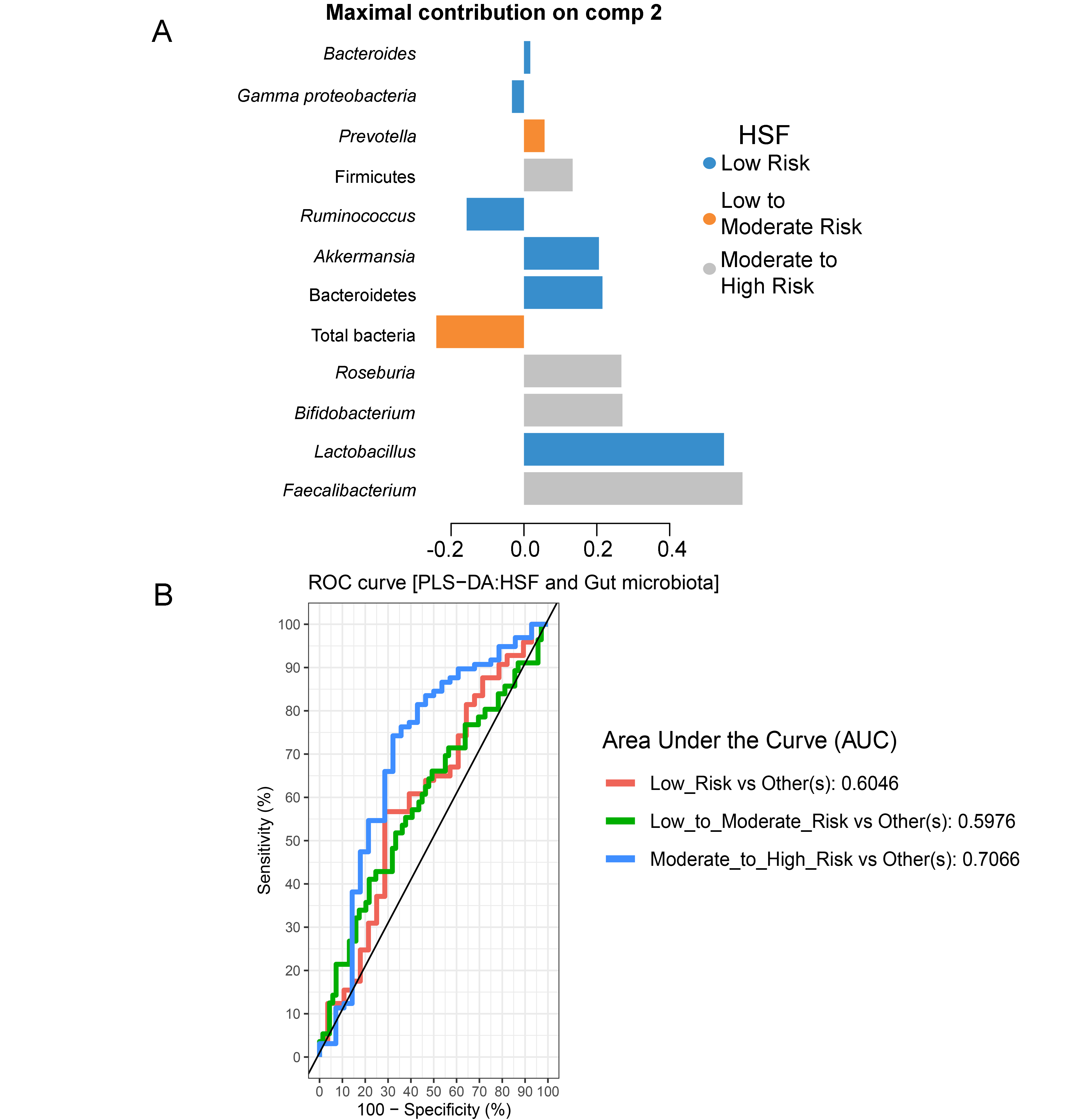

Supplement: Figure S6 — (A) Discriminant analysis demonstrating variable selection (microbiota taxa) for which the median (method = ’median’) is maximum in component 2. Horizontal bars indicate each bacterial taxon assigned to HSF consumption and their length corresponds to the loading weight. The importance of the bacteria contributing to the dimension runs from the bottom to the top of the figure. (B) The plots of ROC curves of PLS-DA classification for gut microbiota in children with HSF consumption in component 2. AUC was calculated and compared between one class versus the others using Wilcoxon test. [file peerj-10-13325-s014.png]

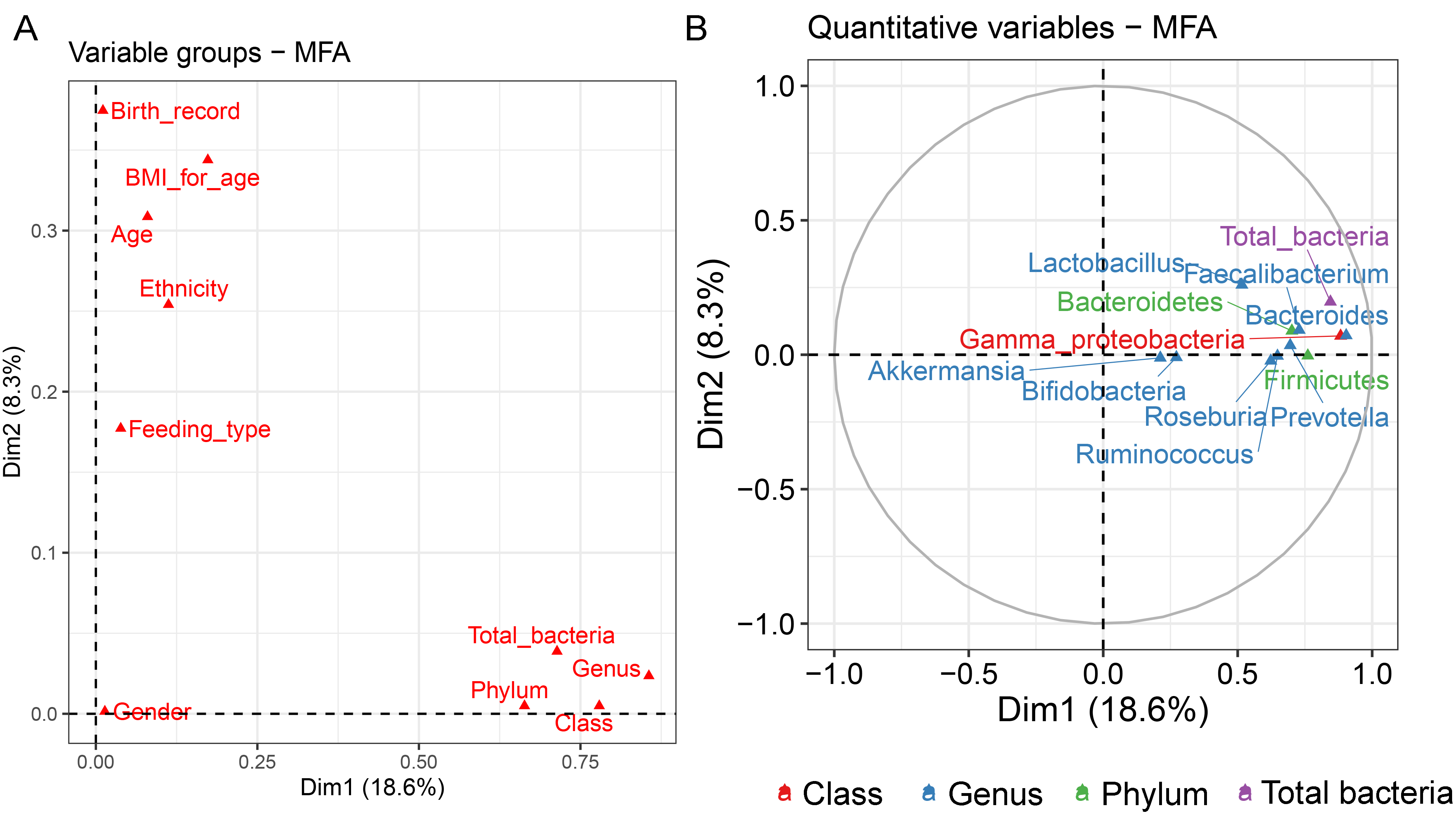

Supplement: Figure S7 — A variable that is close to the circle is highly correlated to the dimension. [file peerj-10-13325-s015.png]

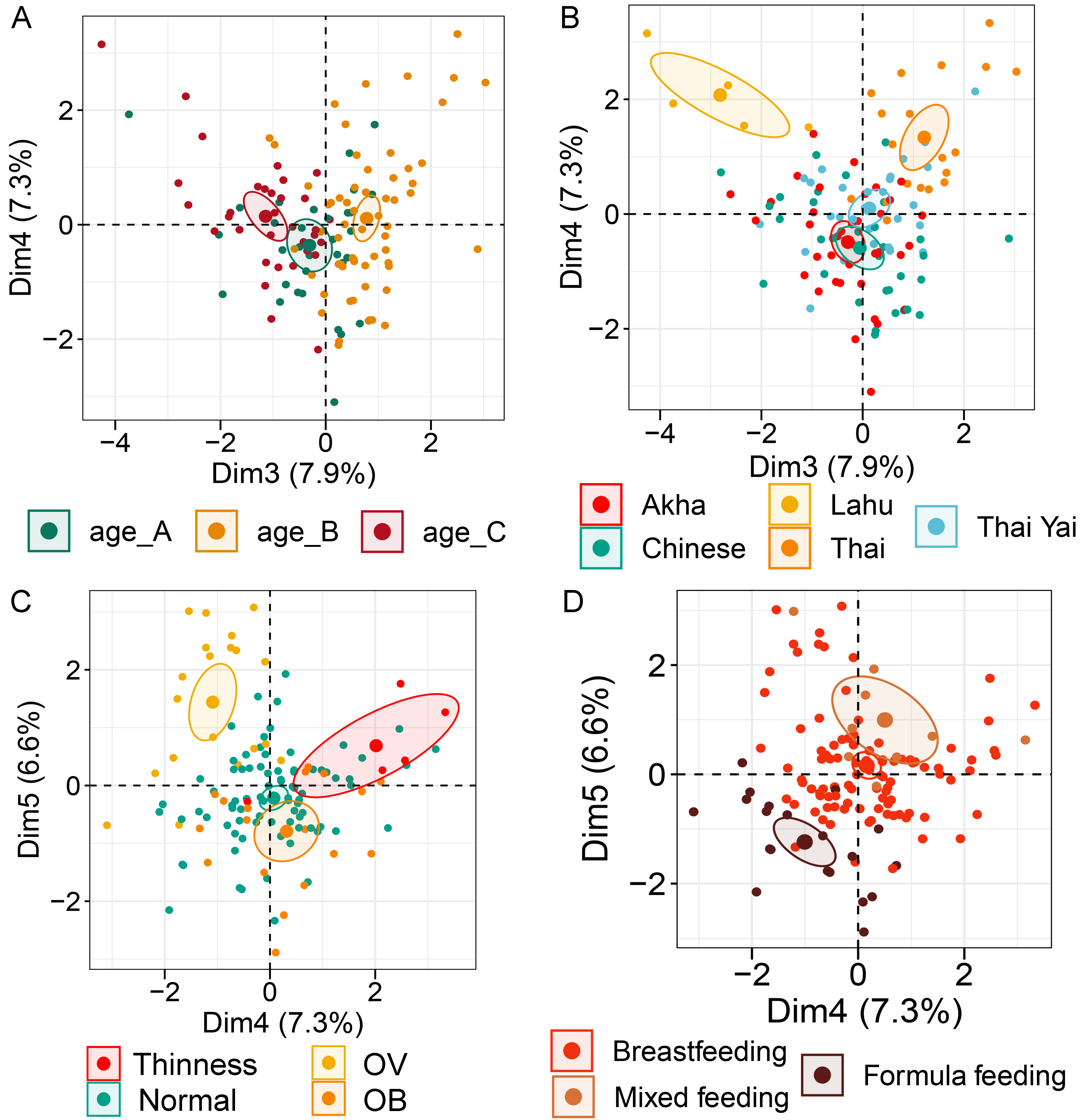

Supplement: Figure S8 — (A) The factor map of individual profiles grouped by age quantile. (B) The factor map of individual profiles grouped by ethnicity. (C) The factor map of individual profiles grouped by BMI z-score. (D) The factor map of individual profiles grouped by feeding type. Individual variables were specified by the 95% confidence ellipses. (A–C) The factor map represents Dim 3 and 4. (D) The factor map for Dim 4 and 5. [file peerj-10-13325-s016.png]

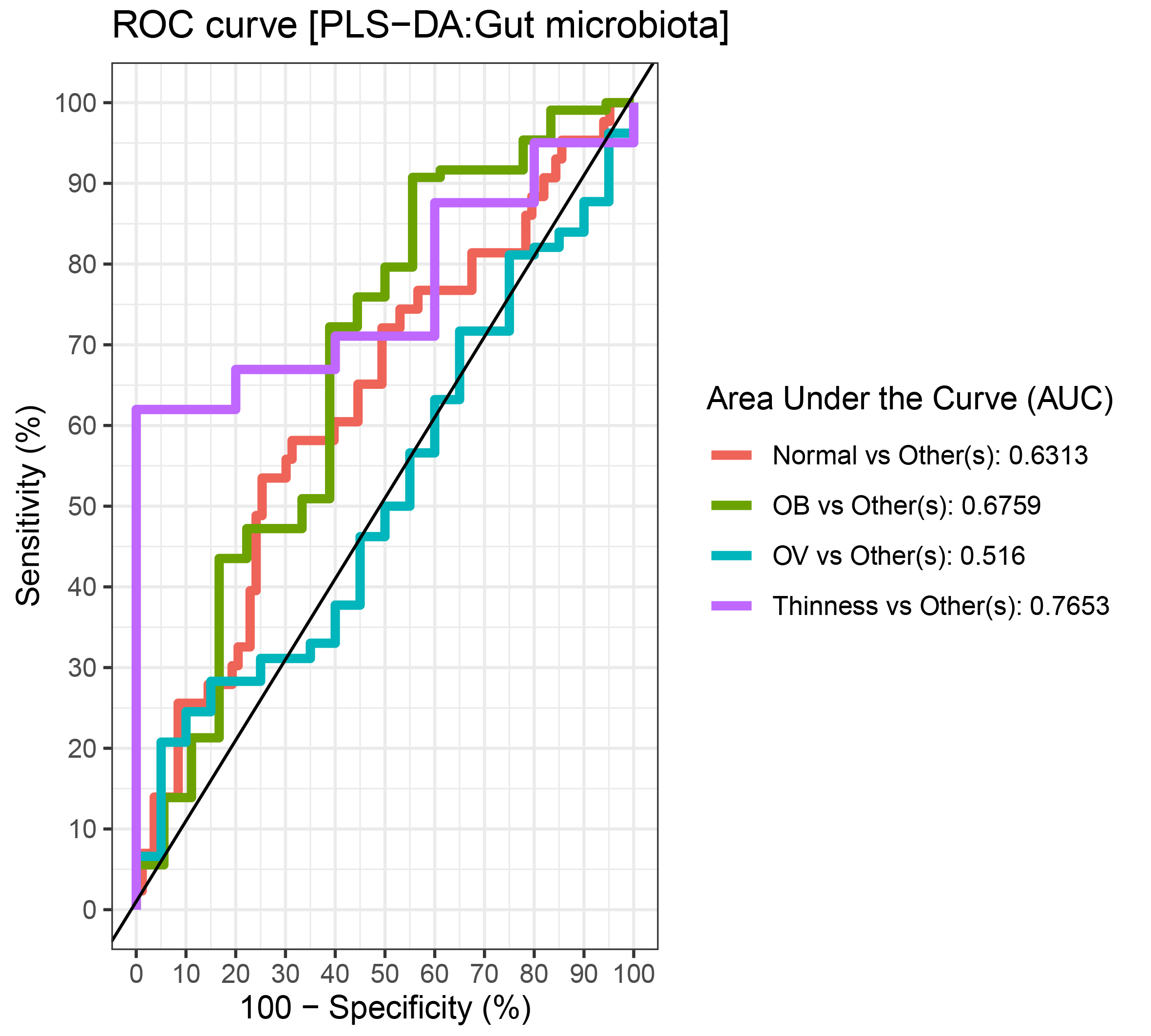

Supplement: Figure S9 — AUC was calculated and compared between one class versus the others using the Wilcoxon test. [file peerj-10-13325-s017.png]

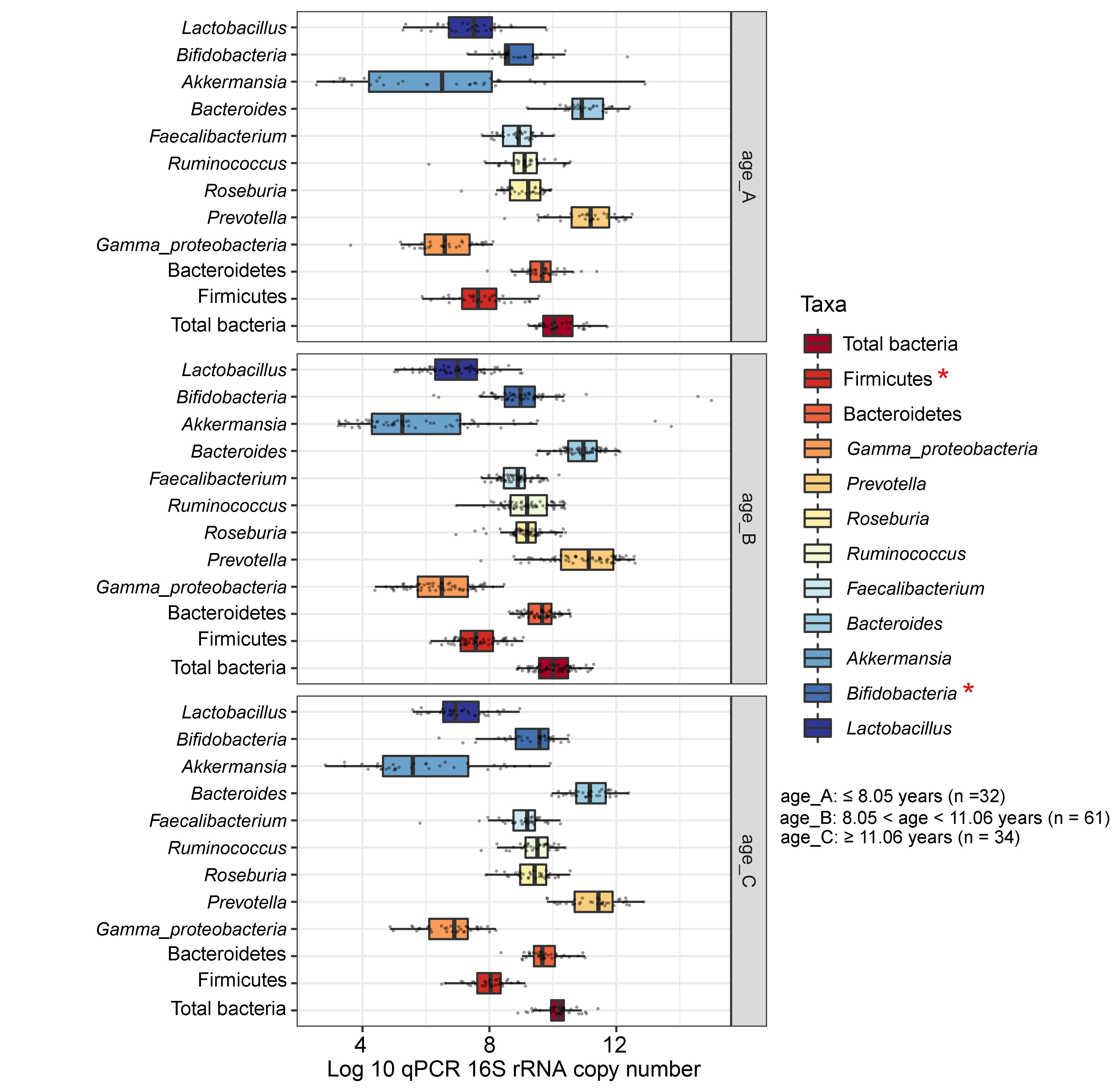

Supplement: Figure S10 — Each subplot is separated by age tertile (corresponding to 25 %, 50 %, and 75 %); age_A ≤ 8.05 years, age_B 8.05 < age < 11.06 years, age_C ≥ 11.06 years. An asterisk indicates a significant difference in microbiota abundance among age tertile (*p < 0.05, ANOVA and Kruskal-Wallis test). [file peerj-10-13325-s018.png]

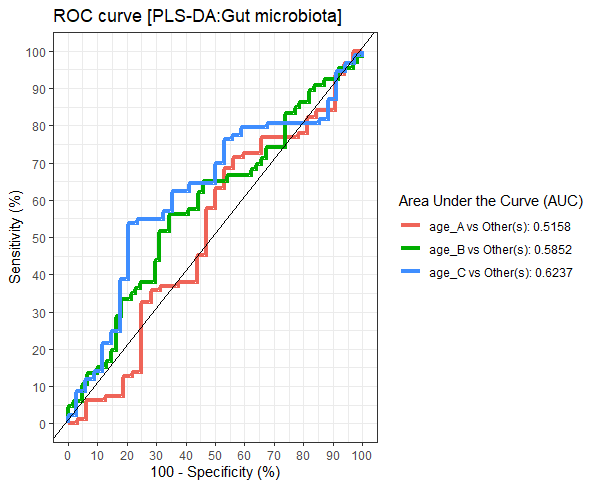

Supplement: Figure S11 — AUC was calculated and compared between one class versus the others using Wilcoxon test. [file peerj-10-13325-s019.png]

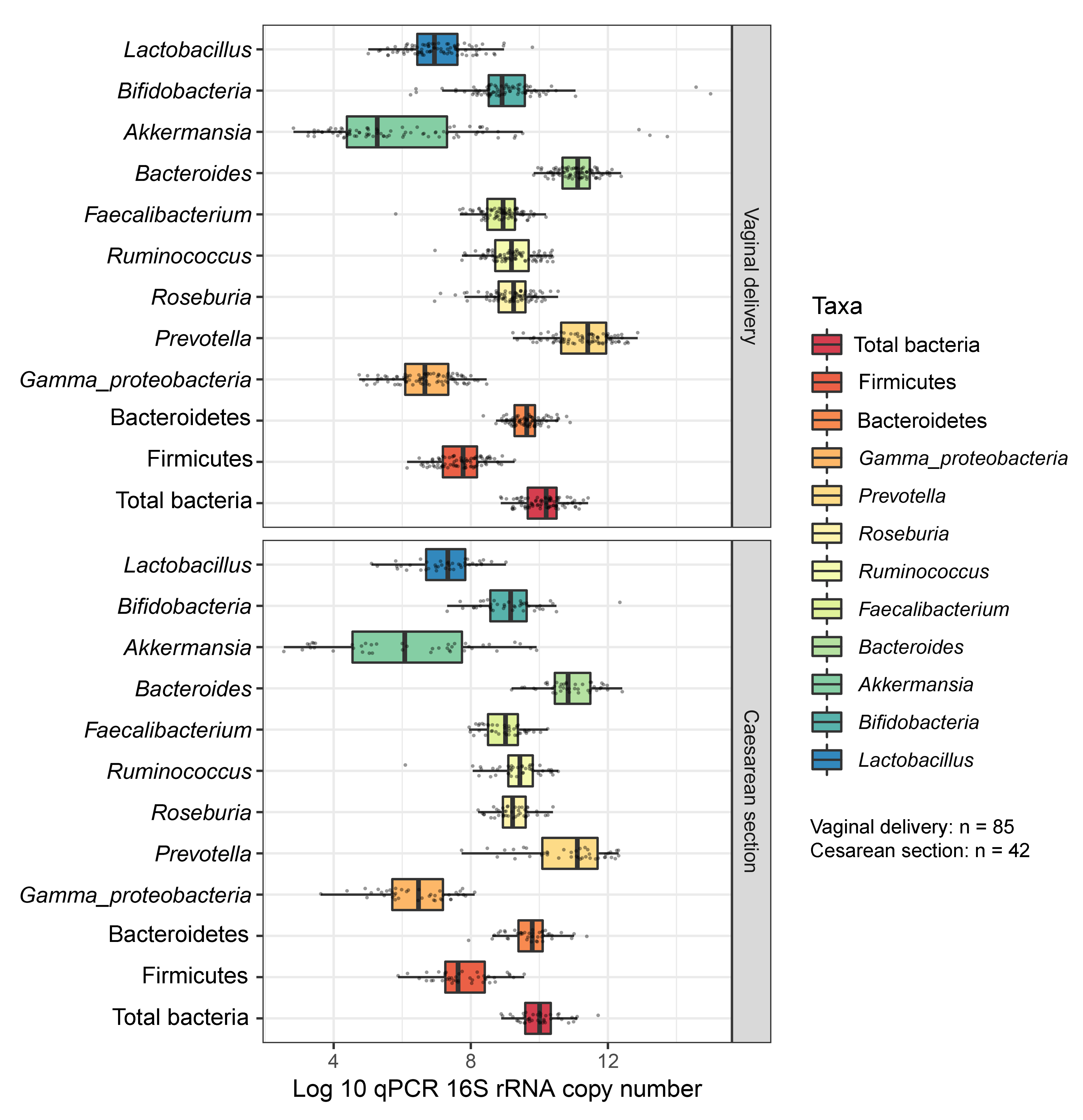

Supplement: Figure S12 — Each subplot is separated by delivery mode. [file peerj-10-13325-s020.png]

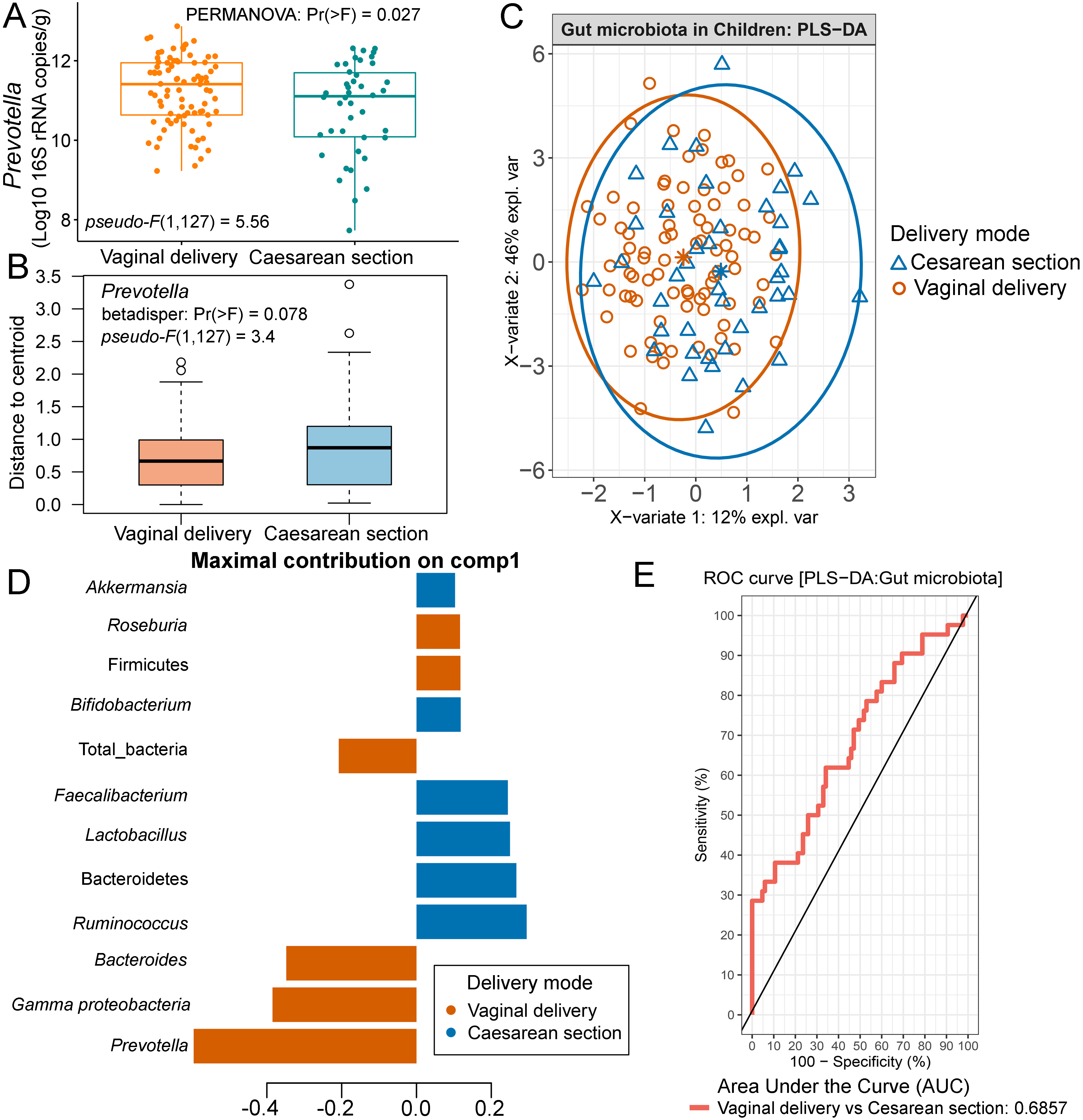

Supplement: Figure S13 — (A) Boxplots showing normalized microbiota abundances based on log10 qPCR 16S rRNA copy number per gram of feces. (B) Boxplots of the distances to centroid for each group. (C) The sample plot represents variations in gut microbiota profiles of children with different birth delivery modes (95% confidence ellipses). An explained variance was based on X-variate (normalized microbiota abundances). (C) Discriminant analysis demonstrating variable selection (microbiota taxa) for which the median (method = ’median’) is maximum in component 1 of the sample plot. Horizontal bars indicate each bacterial taxon assigned to birth delivery mode and their length corresponds to the loading weight. The importance of the bacteria contributing to the dimension runs from the bottom to the top of the figure. (D) The plots of ROC curves of PLS-DA classification for gut microbiota in children with different delivery modes (component 1). AUC was calculated and compared between one class versus the other using Wilcoxon test. [file peerj-10-13325-s021.png]

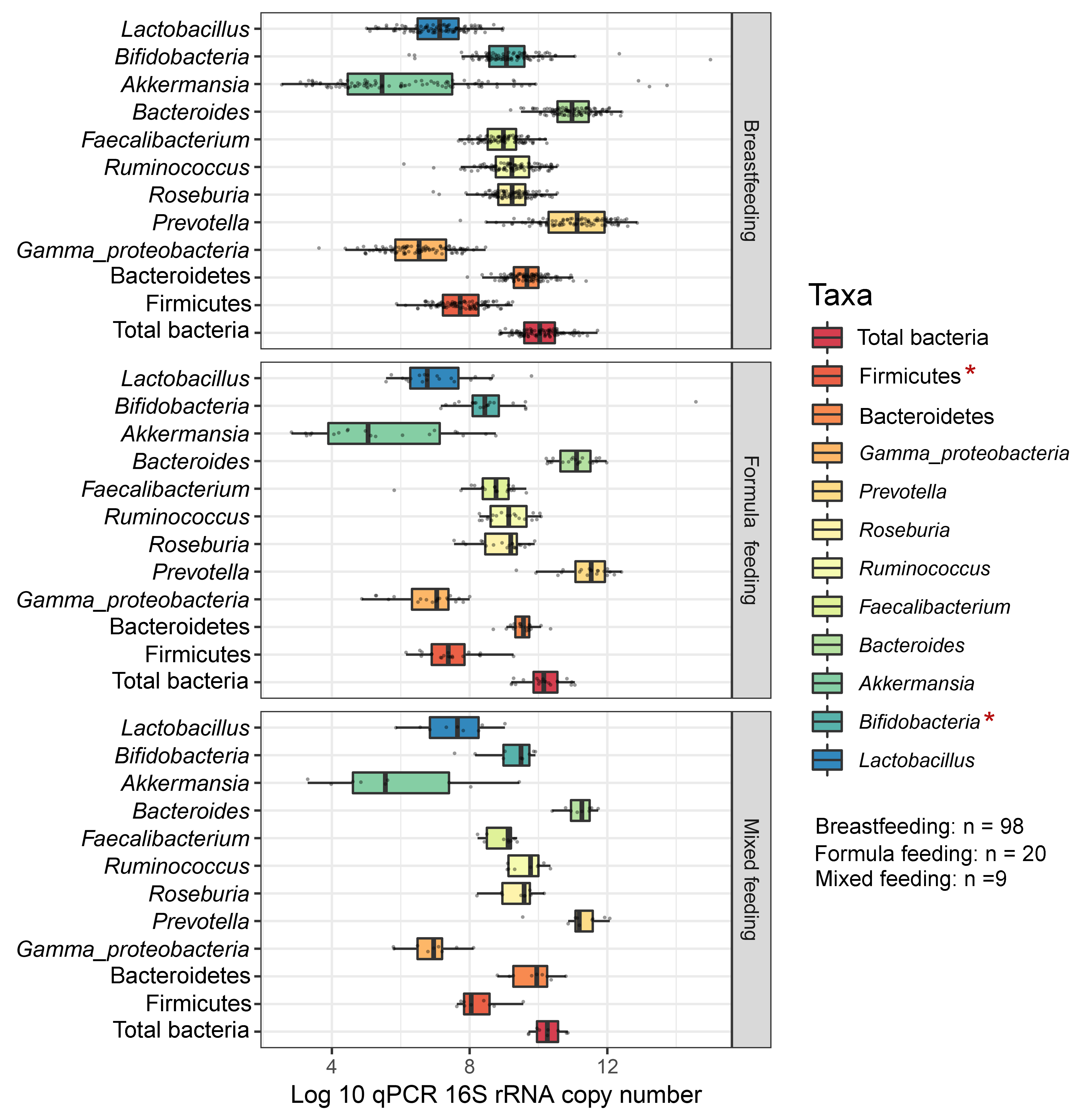

Supplement: Figure S14 — Each subplot is separated by feeding types of children (breast feeding, formula feeding, and mixed feeding). An asterisk indicates a significant difference in microbiota abundance among feeding types (*p < 0.05, ANOVA and Kruskal-Wallis test). [file peerj-10-13325-s022.png]

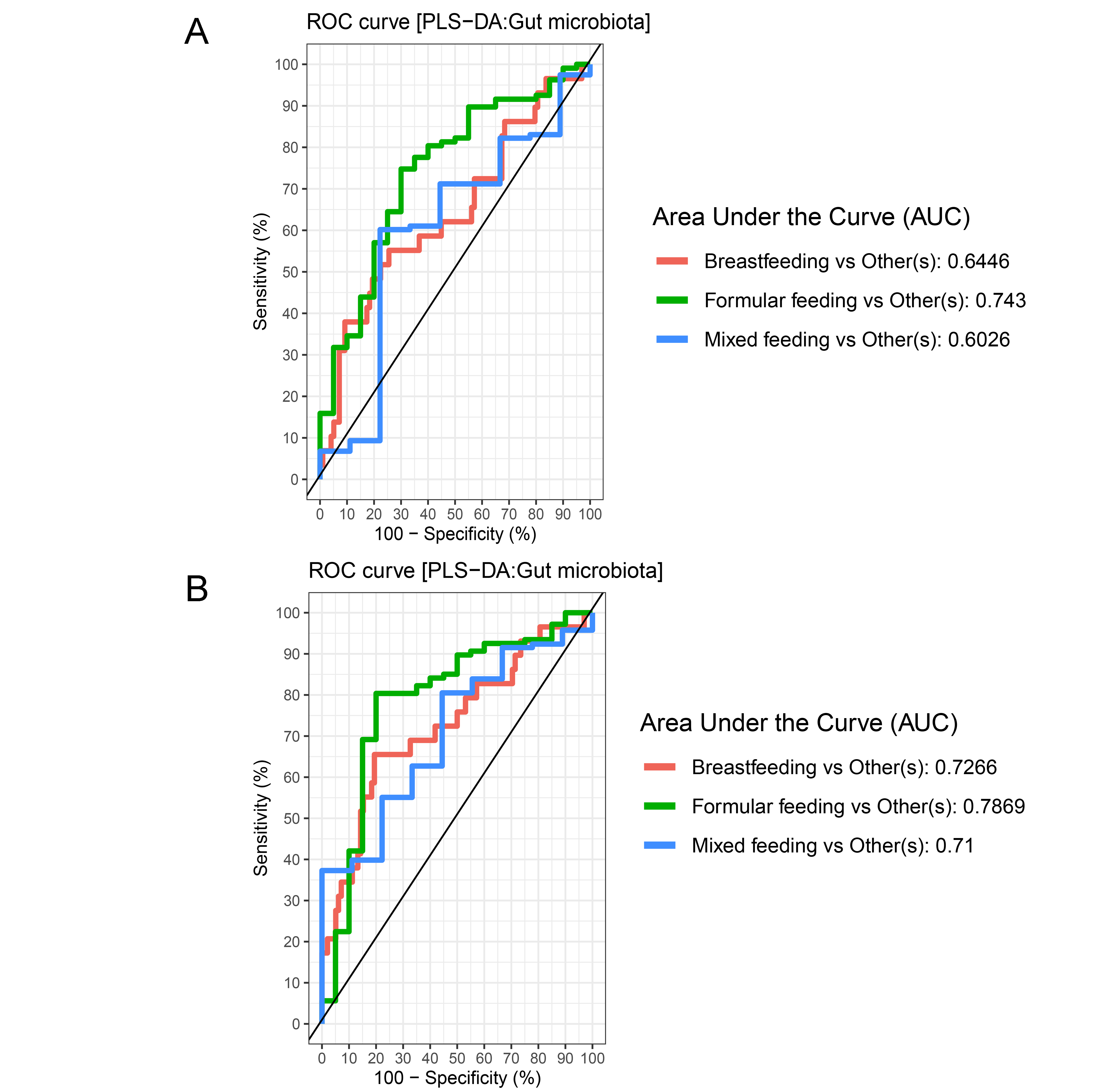

Supplement: Figure S15 — AUC was calculated and compared between one class versus the others using Wilcoxon test. [file peerj-10-13325-s023.png]

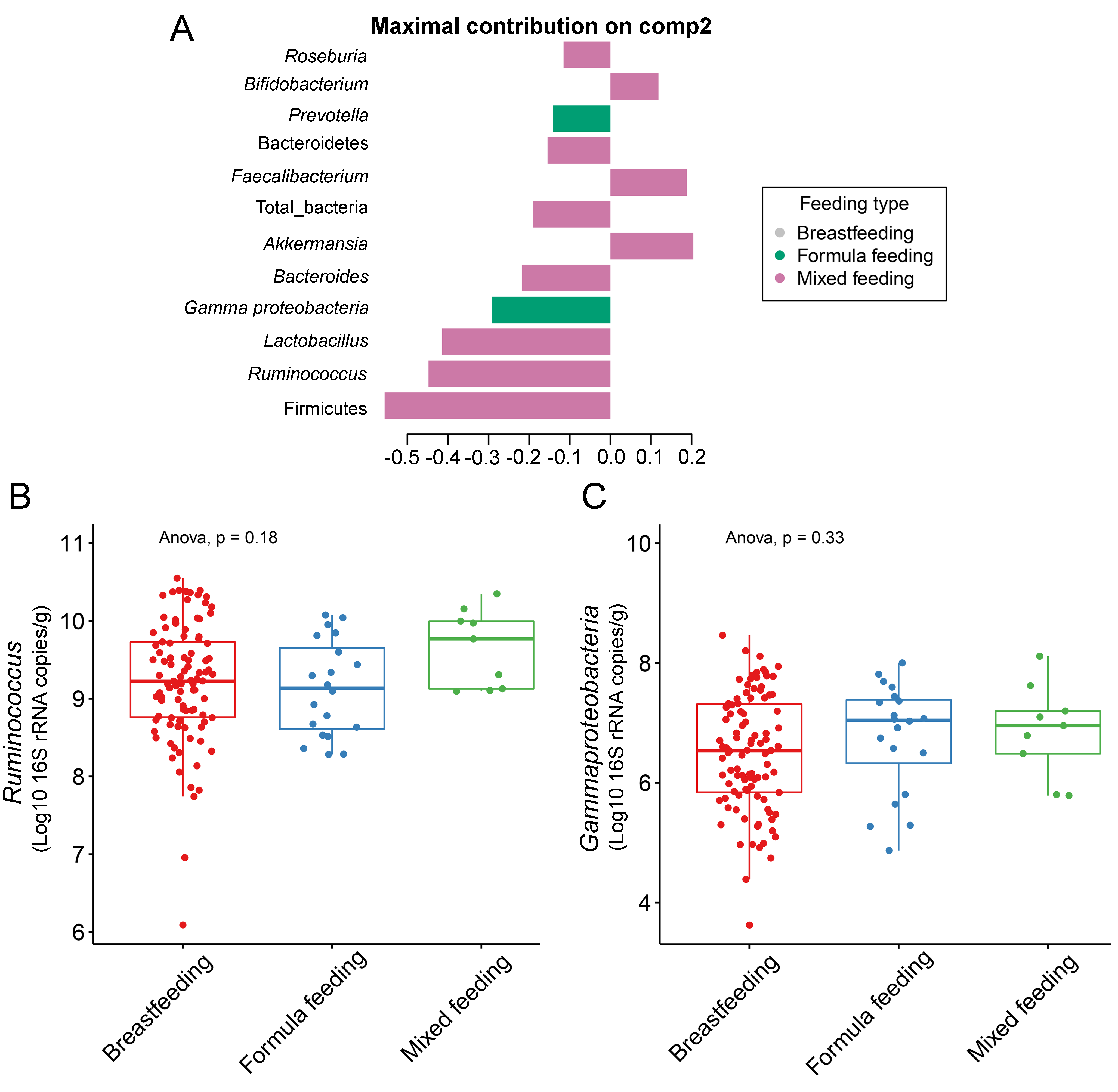

Supplement: Figure S16 — (A) Discriminant analysis demonstrating variable selection (microbiota taxa) for which the median (method = ’median’) is maximum in component 2 of the sample plot. Horizontal bars indicate each bacterial taxon assigned to feeding type and their length corresponds to the loading weight. The importance of the bacteria contributing to the dimension runs from the bottom to the top of the figure. (B–C) Boxplots showing normalized microbiota abundances based on log10 qPCR 16S rRNA copy number per gram of feces. No significant differences in the abundance of Ruminococcus and Gammaproteobacteria among feeding types were found (ANOVA). [file peerj-10-13325-s024.png]

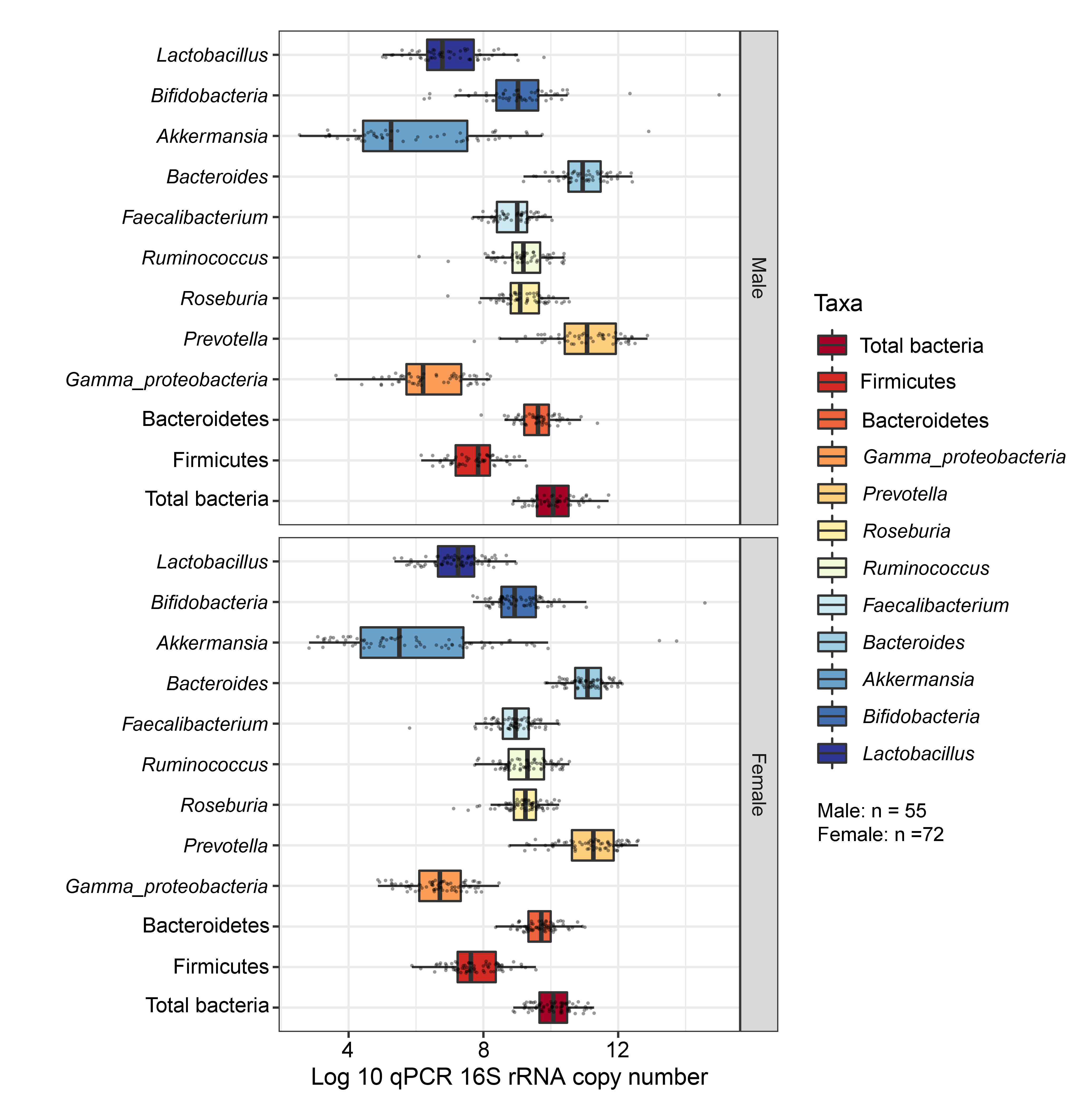

Supplement: Figure S17 — Each subplot is separated by gender. [file peerj-10-13325-s025.png]

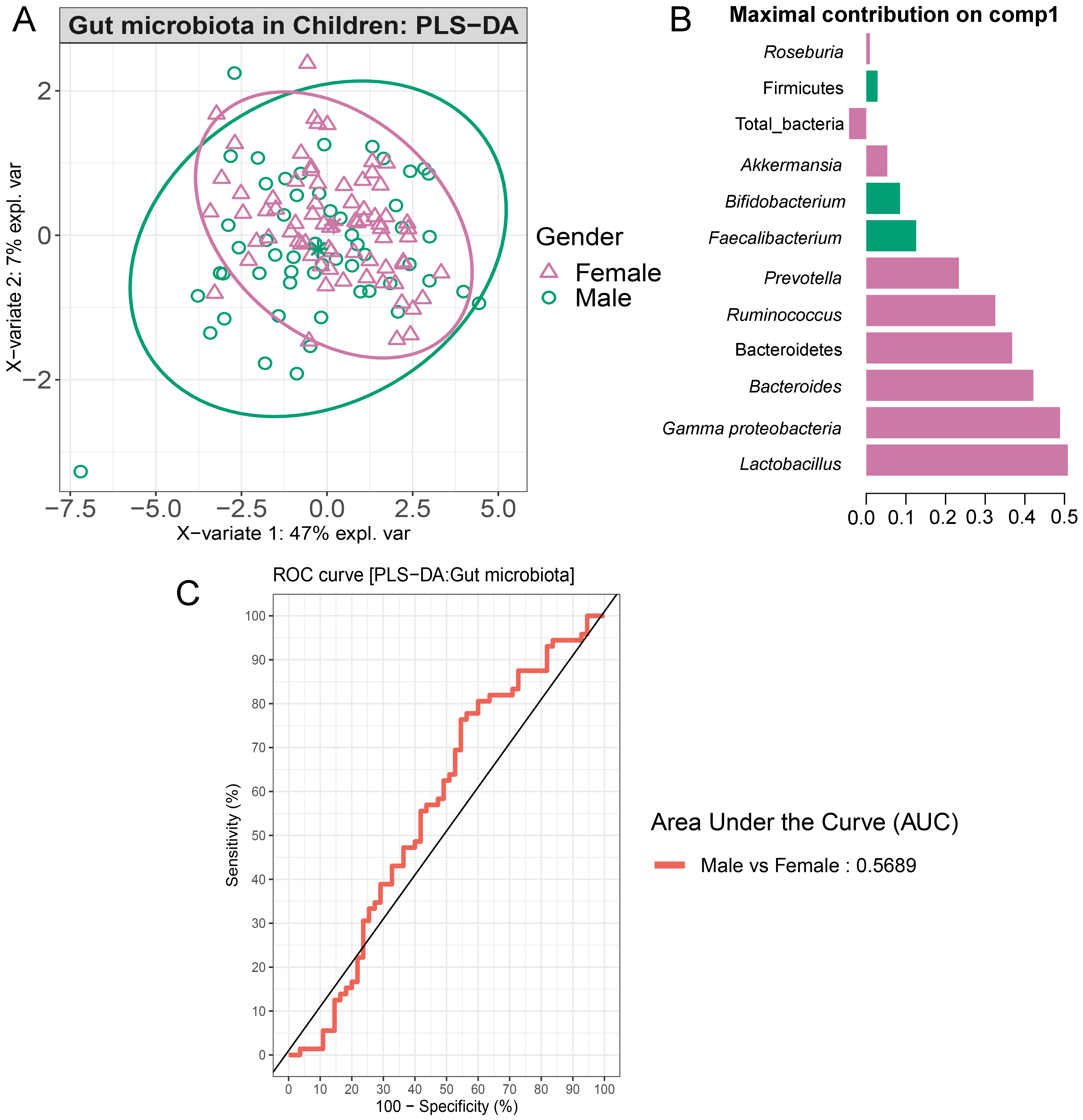

Supplement: Figure S18 — (A) The sample plot represents variations in gut microbiota profiles of school-aged children based on gender (95% confidence ellipses). An explained variance was based on X-variate (normalized microbiota abundances). (B) Discriminant analysis demonstrating variable selection (microbiota taxa) for which the median (method = ’median’) is maximum in component 1 of the sample plot of gender. Horizontal bars indicate each bacterial taxon assigned to gender their length corresponds to the loading weight. The importance of the bacteria contributing to the dimension runs from the bottom to the top of the figure. (C) The plots of ROC curves of PLS-DA classification for gut microbiota in school-aged children based on gender (component 1). AUC was calculated and compared between one class versus the others using the Wilcoxon test. [file peerj-10-13325-s026.png]

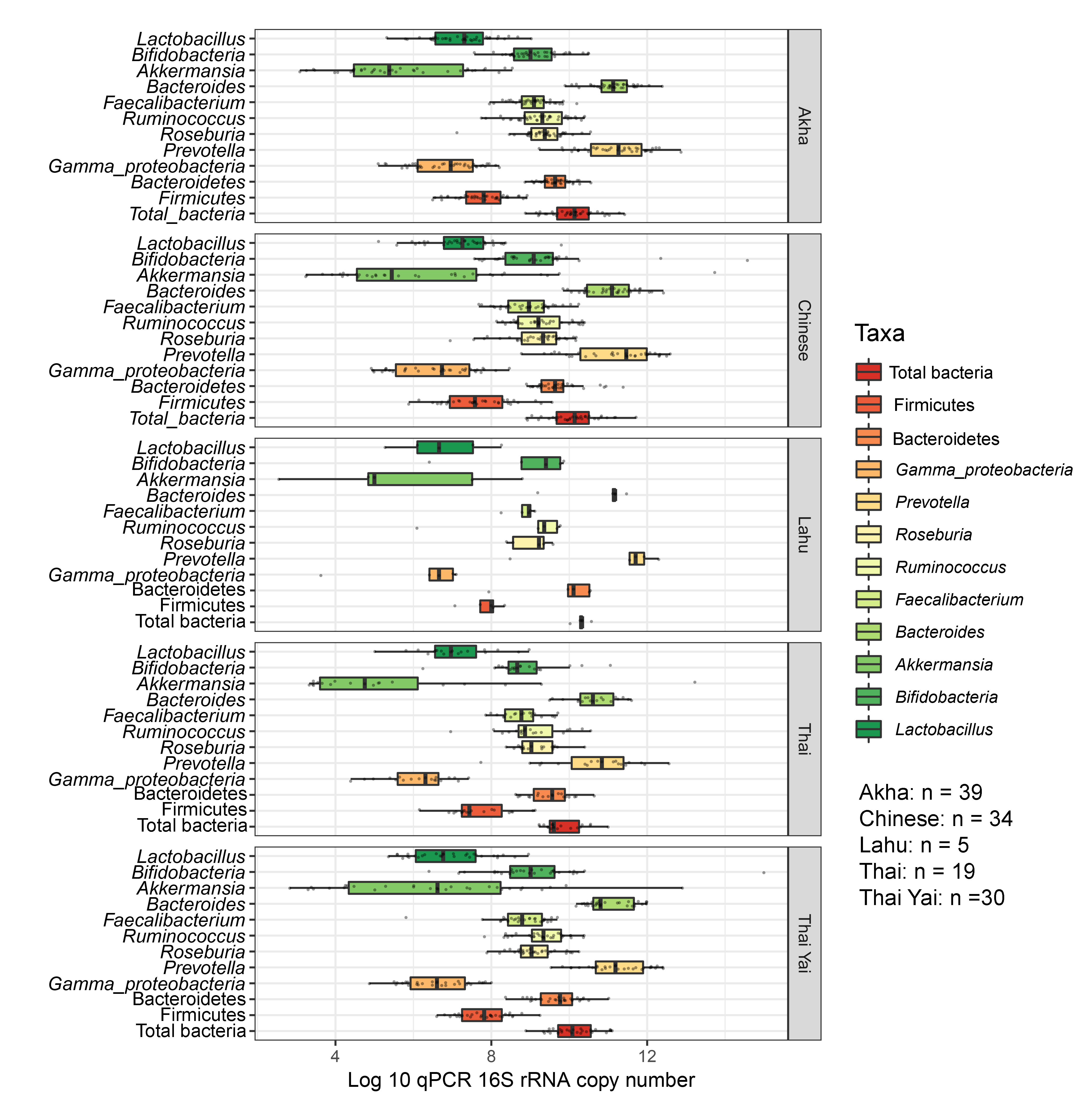

Supplement: Figure S19 — Each subplot is separated by ethnicity. [file peerj-10-13325-s027.png]

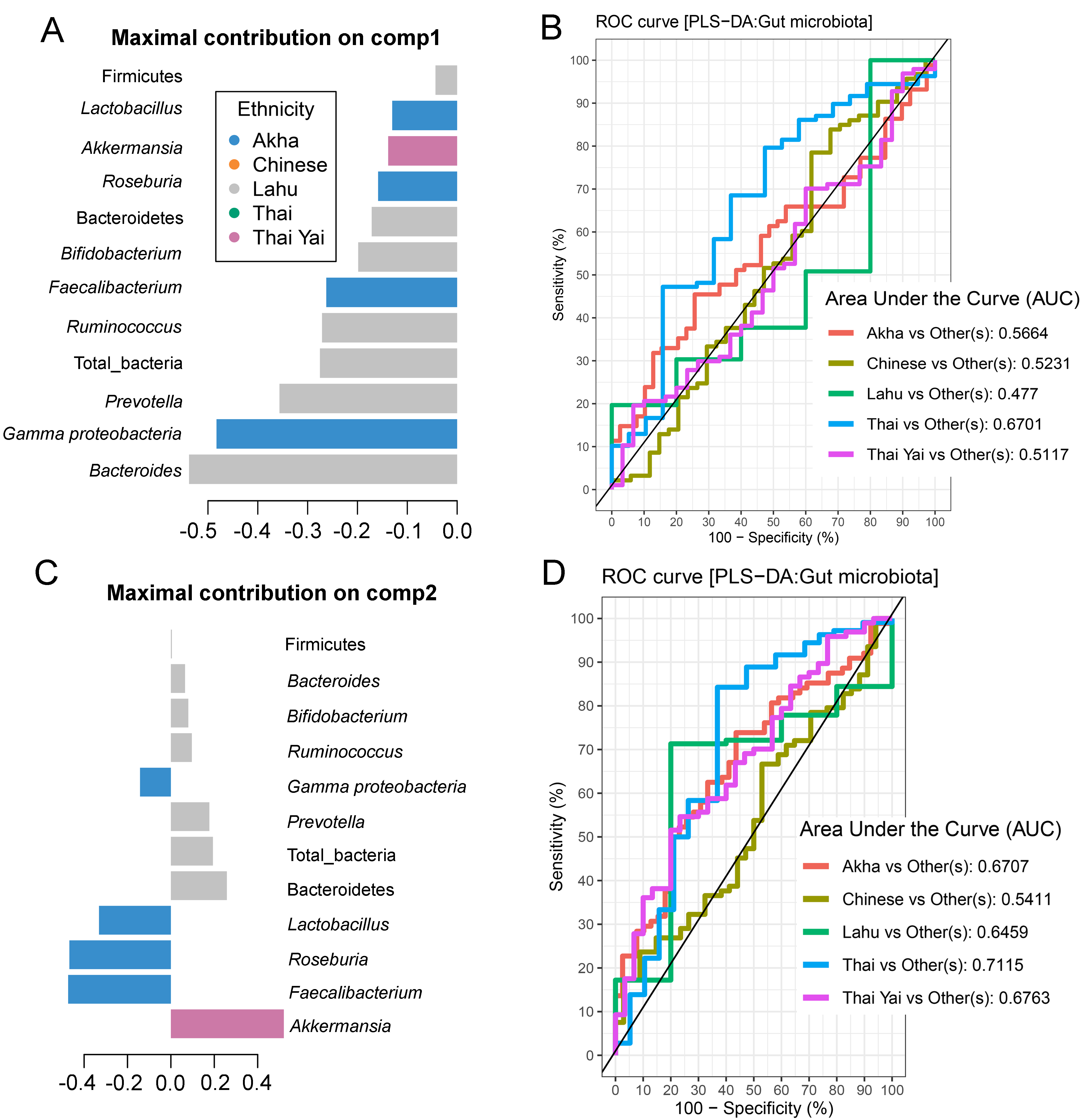

Supplement: Figure S20 — (A and C) Discriminant analysis demonstrating variable selection (microbiota taxa) for which the median (method = ’median’) is maximum in component 1 and 2 of the sample plot of ethnicity. Horizontal bars indicate each bacterial taxon assigned to ethnicity and their length corresponds to the loading weight. The importance of the bacteria contributing to the dimension runs from the bottom to the top of the figure. (B and D) The plots of ROC curves of PLS-DA classification for gut microbiota in school-aged children based on ethnicity (component 2). AUC was calculated and compared between one class versus the others using the Wilcoxon test. [file peerj-10-13325-s028.png]

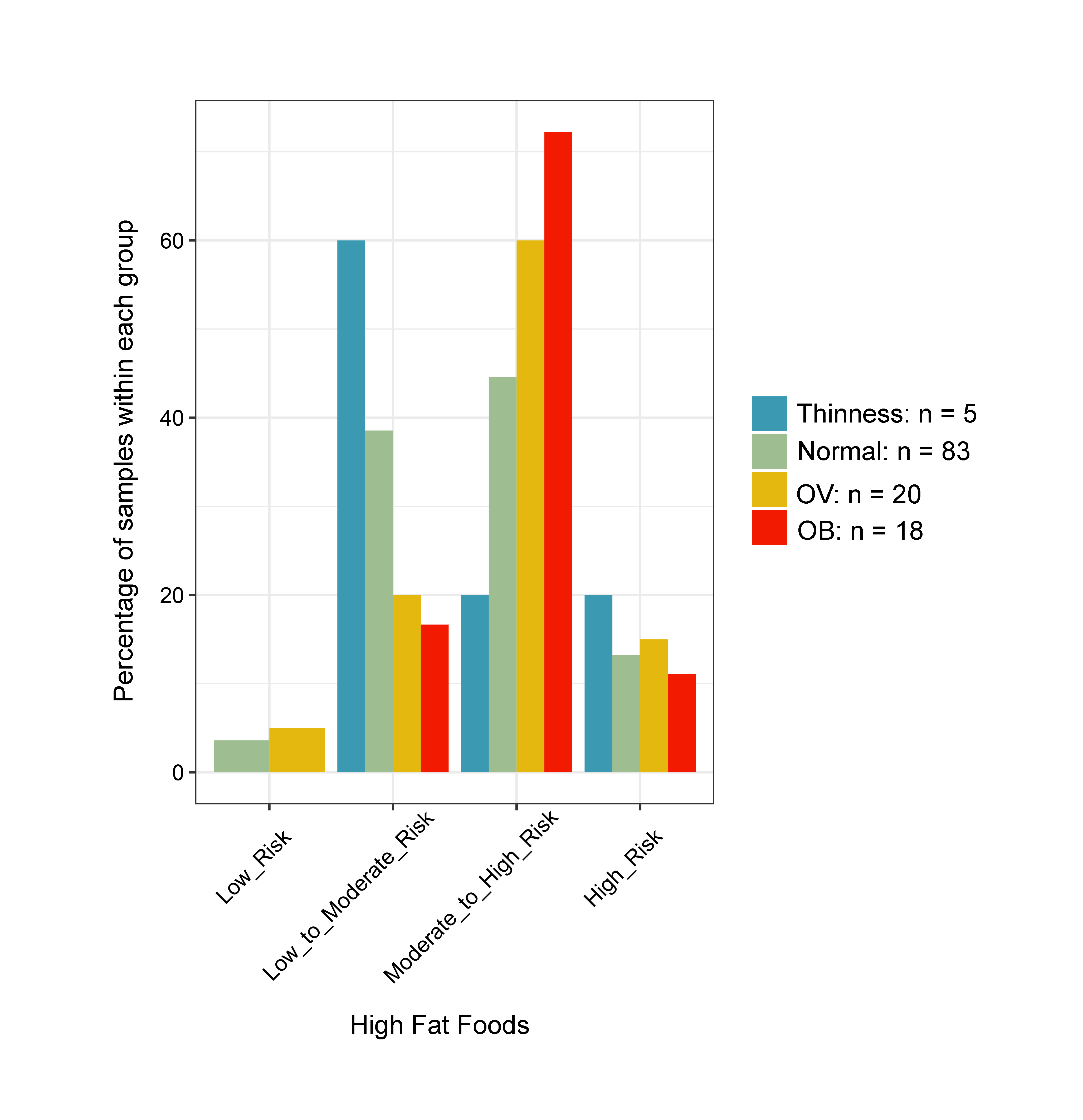

Supplement: Figure S21 [file peerj-10-13325-s029.png]

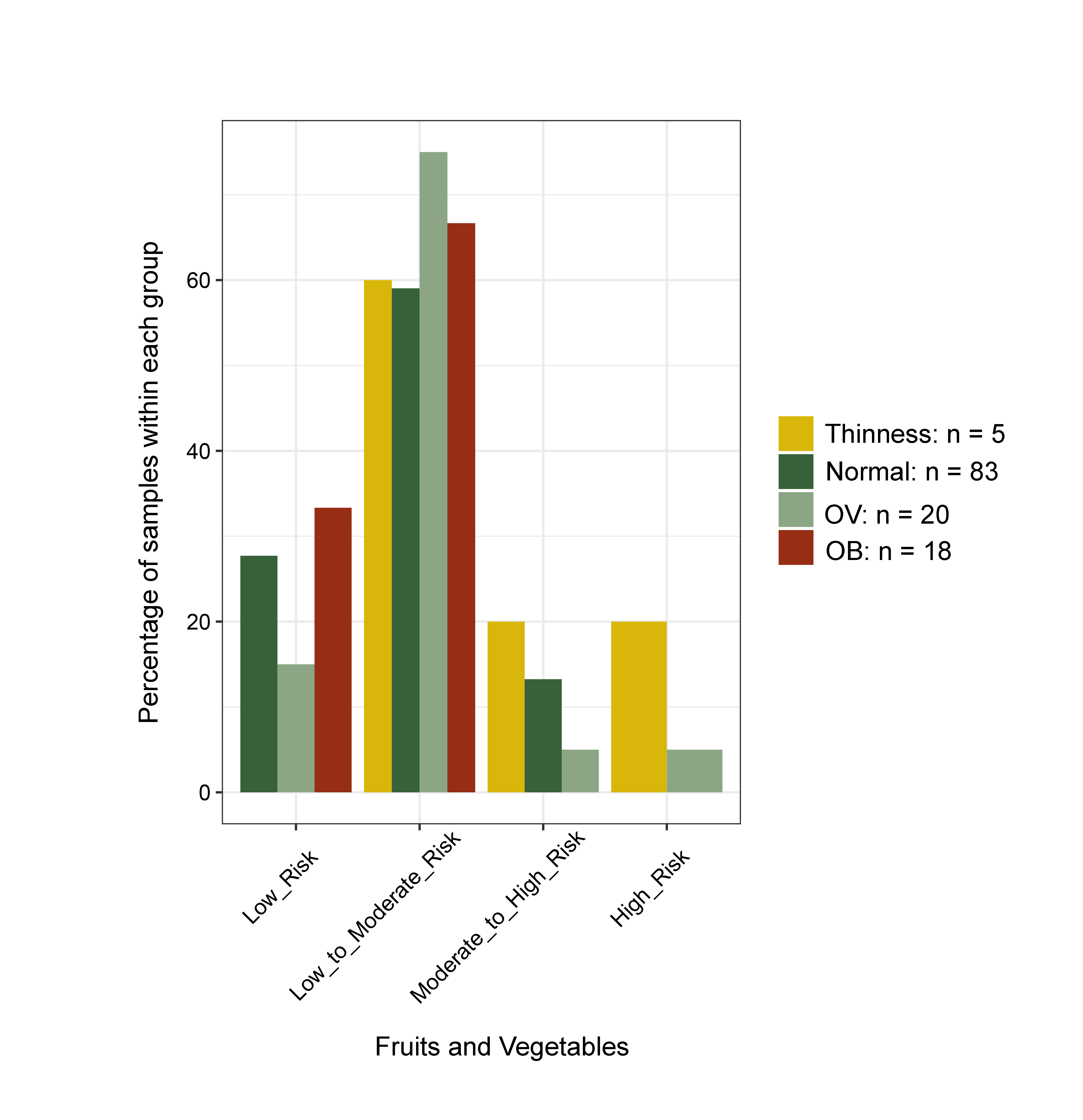

Supplement: Figure S22 [file peerj-10-13325-s030.png]
